# Factors affecting survival and prognosis in surgically treated patients with spinal metastases arising from renal cell carcinoma

**DOI:** 10.1186/s12894-023-01294-7

**Published:** 2023-07-13

**Authors:** Ming Lei, Jun Miao

**Affiliations:** 1 Graduate School, Tianjin Medical University, No. 22, Qi-xiang-tai Road, He-ping District, Tianjin, 300070 China; 2 Department of Orthopedics, Tianjin Hospital, Tianjin University, No.406, Jie-fang South Road, Hexi District, Tianjin, 300210 China

**Keywords:** Renal cell carcinoma, Spinal metastasis, Spine surgery, Prognostic factors, Survival analysis

## Abstract

**Background:**

The objective of this study was to explore the prognostic factors for renal cell carcinoma (RCC) patients with spinal metastasis who underwent surgical treatment in our hospital.

**Methods:**

We retrospectively analyzed the clinical data and survival status of 49 patients with spinal metastases arising from RCC. All patients with spinal metastases underwent surgical treatment. We analyzed a range of factors that may affect the prognosis of patients with RCC. Using Kaplan-Meier method to perform univariate analysis of the factors that might affect spine metastasis free survival (SMFS)and survival after spinal metastasis (OS) respectively. Establish Cox proportional hazards model to extract independent prognostic factors for SMFS and OS.

**Results:**

The mean time of SMFS was 27 months (median 8, range 0–180 months). The mean time of OS was 12.04 months (median 9, range 2–36 months). RCC with visceral metastasis (p = 0.001,HR 11.245,95%CI 2.824–44.776) and AJCC RCC Stage (p = 0.040,HR 2.809,95%CI 1.046–7.543) can significantly affect SMFS. Furthermore, WHO/ISUP Grade (p < 0.001, HR 2.787,95%CI 1.595–4.870), ECOG Score (p = 0.019, HR 0.305,95%CI 0.113–0.825) and multiple spinal metastases (p < 0.001, HR 0.077,95%CI 0.019–0.319) have significant effects on OS.

**Conclusions:**

RCC with visceral metastasis and AJCC RCC Stage were independent prognostic factors for SMFS. WHO/ISUP Grade, ECOG Scores and multiple spinal metastases were independent prognostic factors for OS.

## Background

Renal cell carcinoma (RCC) is the most common type of renal malignancy. Although most renal cancers can be diagnosed early by imaging examination and can be cured by surgical resection, there are still about 30% of patients have recurrence after surgical resection, and 20–40% have distant metastases [1, 2]. Approximately 30% of patients with metastatic renal cell carcinoma (mRCC) develop bone metastases,40% of which occur in the spine.

When patients with RCC develop spinal metastases, their median survival time is about 10 months [3]. Pathological fractures often occur in patients with spinal metastases, resulting in severe pain. Tumor compression of the spinal cord can cause severe neurological dysfunction, such as varying degrees of weakness and even paralysis. These complications severely reduce the patient’s quality of life and lead to a poor prognosis [4].Compared to other spinal metastases, RCC spine metastases tend to be large, highly destructive, and more resistant to systemic therapy and radiation therapy [5].

With the development of new surgical techniques and the continuous progress of implant device design, surgeons can perform increasingly complex surgical operations.

With surgical treatment, doctors can remove the metastatic tumor, reestablish the spinal stability, and relieve spinal cord compression. The patient’s pain is relieved and the nerve function is partially or completely restored. More importantly, surgical treatment provides an opportunity for subsequent systemic treatment.

In recent years, several studies have reported the prognosis and influencing factors of patients with renal cell carcinoma and give some recommendations for treatment opinions. However, only a limited number of previous studies have especially focused on spinal metastases of RCC. The aim of this study is to analyze the clinical data of RCC patients with spinal metastasis who underwent surgical treatment in our hospital, and to explore the potential prognostic factors of RCC patients with spinal metastasis.

## Materials and methods

### Patients

From January 2016 to December 2021, a total of 552 patients were diagnosed with renal cell carcinoma at our hospital, including 83 patients with metastatic renal cell carcinoma. Of these 82 patients with metastatic RCC, 62 patients developed spinal metastases, and 49 of these patients underwent spinal surgery. We reviewed the clinical data of these 49 patients with spinal metastases arising from RCC who accepted surgical intervention in our hospital. This study was approved by the hospital ethics committee, and informed consents were obtained from the participants.

We performed a detailed review and analysis of the patient’s medical records, images, and pathology reports. All patients were followed up every 3 months for the first 6 months after surgery, then every 6 months for the next 2 years, and annually thereafter. All patients were all followed up until December 2021 or until death. The final statuses (died of disease/alive with disease) were acquired through telephone interviews.

### Treatment

All patients received surgical interventions including total en-bloc spondylectomy (TES) or separation surgery. Surgery indications are as follows: (1) Pathological fractures and neurological dysfunction due to spinal metastasis (2) The estimated preoperative life expectancy is greater than 3 months. Personalized surgical strategies were determined according to Tomita, revised Tokuhashi, and Spinal Instability Neoplastic Score (SINS). After surgery, all tumors resected from surgery were sent for pathological examination. Targeted therapy was given to all patients 1 month after surgery.

### Statistical analysis

A series of clinical factors as follows were analyzed to identify independent variables that could predict prognosis. Potential influencing factors include age, age of RCC, sex, metabolism syndrome, RCC with visceral metastasis, WHO/ISUP grade of RCC, metastase location, radical nephrectomy, AJCC RCC Stage, pre-operative Eastern Cooperative Oncology Group(ECOG) score, pre-operative Frankel score, pre-operative KPS score, neutrophil-to-lymphocyte ratio(NLR); platelet-to-lymphocyte ratio(PLR); hemoglobin and albumin levels and lymphocyte and platelet counts(HALP); spinal metastases combined with visceral metastasis; and strategy of spinal surgery(TES surgery/separation surgery).The Tomita, revised Tokuhashi and SINS scores were ruled out because they are considered to have similar or combined significance with the factors above. The optimal cut-off values of NLR、PLR、HALP were determined by X-tile 3.6.1 software 20 (Yale University, New Haven, CT, USA) The optimal cut-off values of age and RCC age were according to their median values.

Spine metastasis free survival (SMFS) was defined as the date from RCC diagnosis to spine metastasis. OS was measured as the date from spine metastasis to cancer-related death, or December 2021. The Kaplan-Meier method was applied to perform the univariate analysis of the factors that might affect SMFS and OS, respectively.

The Cox proportional hazards model was established to extract the independent prognostic factors from factors with p value < 0.1 and identify independent prognostic factors in SMFS and OS, respectively. A p value (two-sided) of < 0.05 was considered significant. All analyses were carried out using SPSS for Windows, version 25.0.0 (SPSS, IBM corp. New York, USA).

## Results

A total of 38 men and 11 women with a mean age of 57.12 (median 58, range 37–75) years were retrospectively reviewed (detailed in Table 1). Mean Age of RCC was 55.18 (median 55, range 35–75) years. Of all patients, 25 underwent radical nephrectomy, the mean SMFS was 27(median 8, range 0–180 months) months. The locations of spinal metastatic lesions were noted, including 40 on the thoracic spine,9 on the lumber spine. Among them,13 patients had multi-level spinal metastases. In our cohort, 9 patients had brain metastases, 4 patients had liver metastases, 7 patients had lung metastases, and 12 patients had two or more sites of metastases.16 patients received separation surgery while the others underwent total en-bloc spondylectomy (TES). After surgery, all patients’ symptoms were alleviated to varying degrees (detailed in Table 1).

**Table 1:** Clinical data of 49 patients with RCC spine metastases

Age(year)	Mean 57.12(9.722)	Median 58	Range 37–75
Age of RCC (year)	Mean 55.18(10.039)	Median 55	Range 35–75
Sex (M / F)	38	11	
SMFS (month)	Mean 27(39.627)	Median 8	Range 0–180
OS (month)	Mean 12.04(8.098)	Median 9	2–36
Metabolism syndrome (+/−)	31/18		
WHO/ISUP	2	16	32.65%
	3	21	42.86%
	4	12	24.49%
Radical nephrectomy	25	(55.10%)	
metastasis location	T	40	81.63%
	L	9	18.37%
	Multiple	13	26.53%
ECOG	Mean 2.78(1.141)	Median 3	Range 1–4
Tomita	Mean 5.24(1.521)	Median 5	Range 3–8
Revised Tokuhashi	Mean 9.71(2.273)	Median 10	Range 6–14
SINS	Mean 10.63(2.455)	Median 11	Range 6–15
TES/Separation surgery	33/16		
Frankel		Pre	Post
	A	5	0
	B	5	3
	C	20	3
	D	16	11
	E	3	32

Abbreviations: RCC,Renal cell carcinoma ; SMFS,Spine metastasis free survival;OS,Overall survival; WHO,World Health Organization; ISUP,International Society of Urology and Pathology,ECOG,Eastern Cooperative Oncology Group; SINS,Spinal Instability Neoplastic Score; TES,total en-bloc spondylectomy;

### Univariate and multivariate analysis of prognostic factors for SMFS

The 1-year,3-year, and 5-year SMFS rates were 55.3% ± 7.6%,45.3% ± 8.2% and 27.9% ± 7.9% respectively, with a mean SMFS of 27 ± 38.38 months (95%CI 15.62–51.7). Potential prognostic factors were listed in Table 2.

**Table 2:** Univariate and multivariate analysis of Spine Metastasis Free Survival (SMFS).

Factors		Univariate				Multivariate				
		Cases	Events	Χ2	p-value	B		HR	95% CI	p-value
Sex	M	38	26	0.436	0.509	NI				
	F	11	7							
Age of RCC	≤ 55	25	17	2.205	0.138	NI				
	> 55	24	16							
Metabolism syndrome	Yes	31	23	0.013	0.911	NI				
	No	18	10							
RCC with visceral metastasis	Yes	23	16	28.4	< 0.001	2.420		11.245	2.824–44.776	0.001
	No	26	17							
RCC NLR	≤ 2.61	22	13	0.064	0.800	NI				
	> 2.61	27	20							
RCC PLR	≤ 115.26	9	4	0.28	0.597	NI				
	> 115.26	40	29							
RCC HALP	≤ 18.68	7	4	0.58	0.810	NI				
	> 18.68	42	29							
Radical nephrectomy	Yes	25	16	4.377	0.241	NI				
	No	24	17							
AJCC RCC Stage	1,2	17	11	13.164	< 0.001	1.033		2.809	1.046–7.543	0.040
	3,4	32	22							
WHO/ISUP Grade	2	16	10	18.508	< 0.001	0.34	1.045		0.668–2.954	0.37
	3	21	15							
	4	12	8							

Abbreviations: RCC,Renal cell carcinoma ; NLR,neutrophil-to-lymphocyte ratio; PLR,platelet-to-lymphocyte ratio; HALP,hemoglobin and albumin levels and lymphocyte and platelet counts; AJCC,American Joint Committee on Cancer; WHO,World Health Organization; ISUP,International Society of Urology and Pathology;NI,not included in the multivariate analysis; NS,not significant in the multivariate analysis.

Univariate analysis showed that patients with visceral metastasis (p < 0.001) had a significantly lower SMFS, while patients with higher AJCC RCC Stage (p < 0.001) and WHO/ISUP Grade (p < 0.001) of RCC ones had lower SMFS.

Results from multivariate analysis showed that RCC with visceral metastasis (p = 0.001, HR 11.245,95%CI 2.824–44.776) and AJCC RCC Stage (p = 0.040, HR 2.809,95%CI 1.046–7.543) were independent prognostic factors for spinal metastasis.

**Fig. 1:**
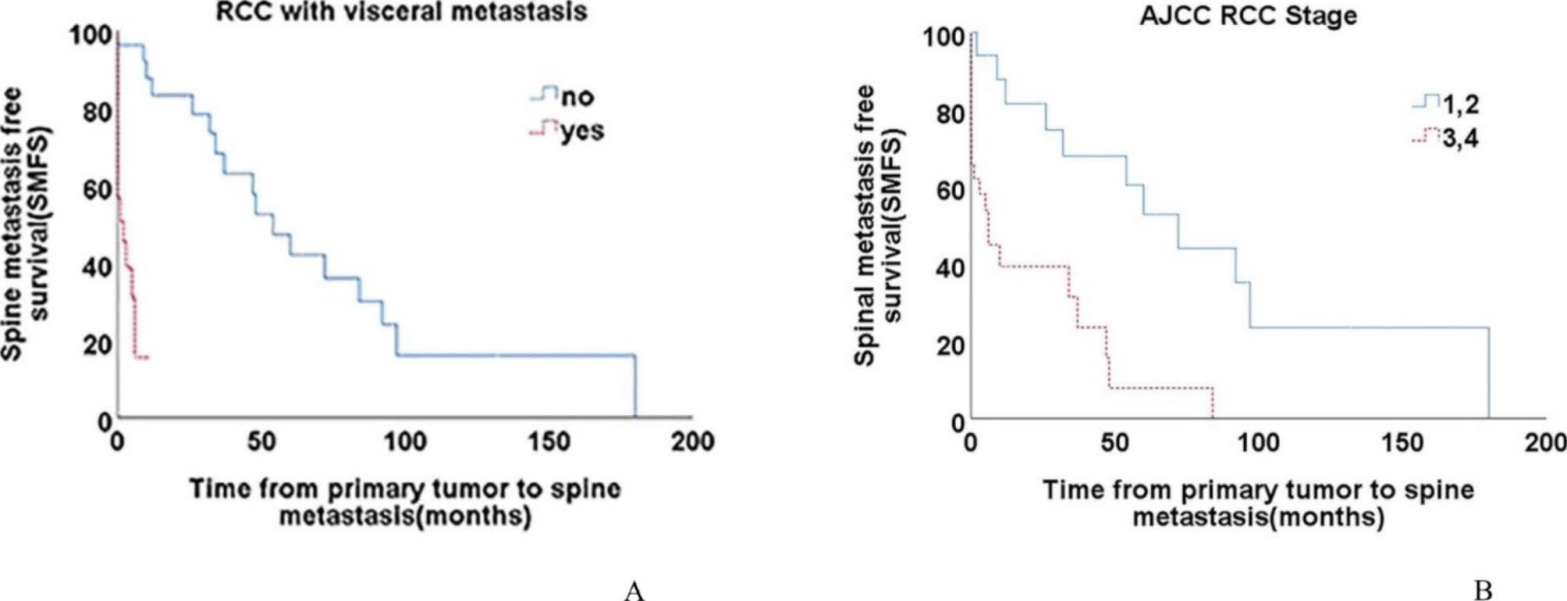
Kaplan-Meier curves of Spine Metastasis Free survival (SMFS) for **(A)** RCC with visceral metastasis **(B)** AJCC RCC stage

However, according to the Cox proportional hazard analysis outcome, WHO/ISUP Grade was not independent prognostic factor for SMFS. The Kaplan–Meier curves of SMFS in RCC with visceral metastasis and AJCC RCC Stage status are shown in Fig. 1A and B.

### Univariate and multivariate analysis of prognostic factors for OS

By the end of the follow-up in December 2021,16 patients died of the disease. The 3-month,6-month,1-year,and 2–year OS rates were 91.8% ± 3.9%,83.3% ± 5.4% ,59.4 ± 8.2% and 17.5% ±7% and respectively, with a median OS was 12.04 ± 8.10 months (95%CI 9.71–14.37). Potential prognostic factors affecting OS are shown in Table 3.

**Table 3:** Univariate and multivariate analysis of Overall Survival (OS).

Factors		Univariate				Multivariate			
		Cases	Events	Χ2	p-value	B	HR	95% CI	p-value
Sex	M	38	26	2.332	0.127	NI			
	F	11	7						
Age of RCC	≤ 55	25	17	1.527	0.217	NI			
	> 55	24	16						
Age of spinal metastases	≤ 58	26	17	1.523	0.217	NI			
	> 58	23	16						
Metabolism syndrome(yes/no)	Yes	31	23	0.94	0.759	NI			
	No	18	10						
Spinal metastases combined with visceral metastasis (yes/no)	Yes	32	18	5.914	0.015	0.366	1.442	0.41–5.702	0.569
	No	17	15						
NLR	≤ 7.78	38	26	0.460	0.497	NI			
	> 7.78	11	7						
PLR	≤ 282.29	42	27	3.563	0.059	NI			
	> 282.29	7	6						
HALP	≤ 20.67	16	9	5.063	0.024	0.631	1.88	0.695–5.086	0.214
	> 20.67	33	24						
Pre-op Frankel score (A–C/D,E)	A–C	30	22	9.776	0.002	0.453	1.573	0.250–9.900	0.629
	D,E	19	11						
AJCC RCC Stage	1,2	17	11	5.177	0.023	-0.101	0.904	0.250–3.269	0.877
	3,4	32	22						
WHO/ISUP Grade	2	16	10	15.477	< 0.001	1.025	2.787	1.595–4.870	< 0.001
	3	21	15						
	4	12	8						
Pre-op KPS Score (≤ 60/>60)	≤ 60	34	24	8.608	0.003	-0.175	0.84	0.120–5.880	0.86
	> 60	15	9						
ECOG Score (1–2/3–4)	0–2	18	11	11.566	0.001	-1.188	0.305	0.113–0.825	0.019
	3–5	31	22						
multiple spinal metastases	YES	13	10	28.976	< 0.001	-2.563	0.077	0.019–0.319	< 0.001
	NO	36	23						
Strategy of spinal surgery(TES surgery/separation surgery)	TES surgery	33	24	19.027	< 0.001	0.883	2.301	0.518–10.227	0.274
	Separation surgery	16	9						

Abbreviations: NLR,neutrophil-to-lymphocyte ratio; PLR,platelet-to-lymphocyte ratio; HALP,hemoglobin and albumin levels and lymphocyte and platelet counts; AJCC,American Joint Committee on Cancer; WHO,World Health Organization; ISUP,International Society of Urology and Pathology; Pre-op ,preoperative; KPS,Karnofsky Performance Status; ECOG,Eastern Cooperative Oncology Group; TES,total en-bloc spondylectomy;NI,not included in the multivariate analysis; NS,not significant in the multivariate analysis.

Univariate analysis using the Kaplan–Meier method showed that poorer OS was associated with(1)Spinal metastases combined with visceral metastasis (p = 0.015);(2)HALP ≤ 20.67 (p = 0.024);(3)Pre-op Frankel score A–C (p = 0.002);(4)AJCC RCC Stage 3,4(p = 0.023);(5)Higher WHO/ISUP Grade(p < 0.001);(6)Pre-op KPS Score ≤ 60(p = 0.003);(7)Higher ECOG Score(p = 0.001);(8)multiple spinal metastases(p < 0.001);(9)Patients who underwent separation surgery(p < 0.001).

**Fig. 2:**
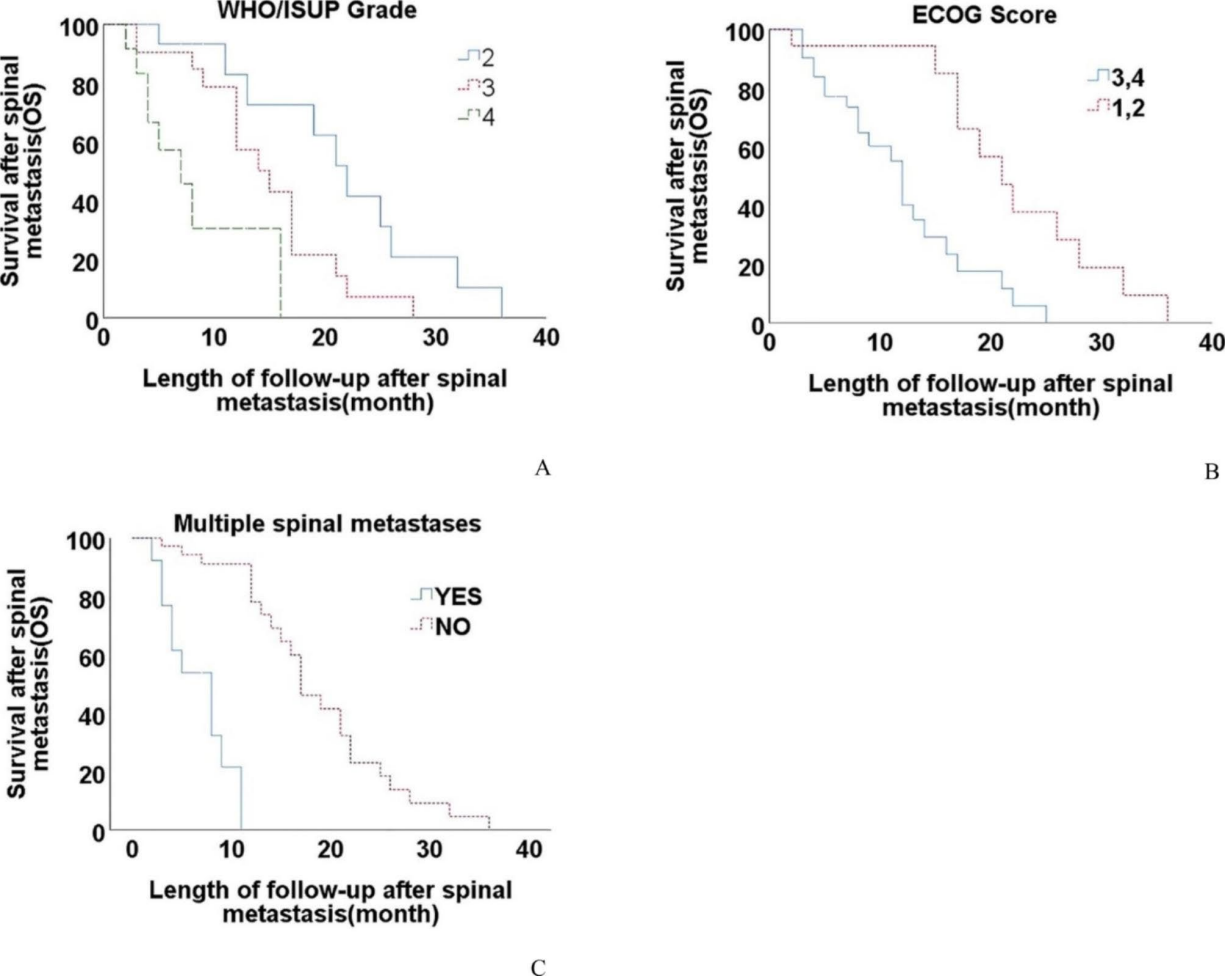
Kaplan-Meier curves of overall survival (OS) for **(A)** WHO/ISUP Grade; **(B)** ECOG Score; **(C)** multiple spinal metastases

Multivariate analysis showed that WHO/ISUP Grade (p < 0.001, HR 2.787,95%CI 1.595–4.870), ECOG Score (p = 0.019, HR 0.305,95%CI 0.113–0.825) and multiple spinal metastases (p < 0.001, HR 0.077,95%CI 0.019–0.319) were independent prognostic factors for OS. The Kaplan–Meier curves of OS using WHO/ISUP Grade, ECOG Score and multiple spinal metastases status are shown in Fig. 2A, B and C.

## Discussion

Patients with metastatic renal cell carcinoma always have a poor survival prognosis [6]. RCC with spinal metastases may cause pathologic fractures and spinal cord compression that seriously affect performance status and quality of life.

In previous medical conditions, surgical treatment plays a very limited role in treating spinal metastases. Surgeons often perform palliative spinal decompression by posterior laminectomy alone to rescue spinal cord function. However, posterior laminectomy decompression cannot achieve complete resection of the metastases and disrupt the stability of the spine. Local recurrence occurs very soon.

With the application of new technologies in the treatment of spinal metastasis, including reconstruction of spinal stability, artificial vertebral replacement, and intraoperative monitoring of spinal cord function. Surgeons are able to perform more precise spinal decompression and reconstruct the spine sequence. As these new techniques are gradually popularized in clinical treatment, surgical treatment outcome for spinal metastases are getting better and better. Increasingly surgeons are being able to provide circumferential decompression and vertebral column reconstruction.

The application of TES surgical techniques in the treatment of spinal metastases was reported in 1994 by Tomita et al. [7]. This surgical technique can provide the best chance of local metastases control, ensure neurologic improvement and spinal stability and extend survival for selected patients. Recommended indications for TES surgery is metastases did not spread into or invade adjacent visceral organs, it showed little or no adhesion to the vena cava or aorta, and it did not show multiple metastases. A contiguous involvement of more than three vertebrae represented a relative contraindication for the TES surgery [8].

When patients with spinal metastasis are complicated with visceral metastases, the systemic condition is poor and the life expectancy is short. “Separation surgery” can relieve spinal cord compression, restore spinal stability, and provide the possibility for subsequent local radiotherapy. Laufer et al. [9] reported this “separation surgery” in which the spinal cord was decompressed by limited posterolateral tumor resection and posterior segmental instrumentation. The operation was performed to remove the lamina and facet joints, the posterior longitudinal ligament and part of the vertebral body to ensure the presence of 5–8 mm decompression area around the spinal cord. Whether to perform the anterior reconstruction is determined by how much of the vertebral body is resected. Many studies have demonstrated that Separation surgery with Stereotactic body radiation therapy (SBRT) for metastatic epidural spinal cord compression (MESCC) was effective in decompression and long-term local control [10–12].

In our work, both of these two surgical strategies were applied and achieved good results. The patient’s preoperative KPS scores were improved (detailed in Table 1). Univariate analysis showed that poorer OS was associated with separation surgery. However, the current study only reported significant impact on OS in the univariate, but not multivariate analysis. It was probably due to selection bias that led to statistically insignificant result. In patients with isolated spinal metastasis without visceral metastases, we often perform TES surgery for a radical resection of the metastases. For patients with discontinuous multiple vertebral metastases, or poor systemic conditions, we prefer to perform the separation surgery.

We analyzed the potential factors that affect SMFS In our study. The result showed that RCC patients with visceral metastasis, higher AJCC RCC Stage and WHO/ISUP Grade can significantly affect the SMFS. The AJCC RCC Stage, was designed based on the TNM staging of renal malignancies. Tumor size is an important component of the TNM staging system, and it is also one of the most important factors in assessing the prognosis of renal cancer [13].Regardless of kidney tumor size, stage 4 was defined if metastasis were present and represents a poor prognosis. WHO/ISUP is a grading system for clear cell renal carcinoma (cRCC)and papillary renal cell carcinoma(pRCC)and it is considered to the most important prognostic factor besides the TNM stage. Studies have shown a strong correlation between WHO/ISUP and 5-year survival in cRCC. For patients with the same tumor stage, the pathological nuclear grade of the tumor was an independent prognostic factor [14, 15]. This conclusion was also confirmed in our study. Multivariate analysis shows that WHO/ISUP grade is an independent prognostic factor for OS.

Multiple spine metastasis is another independent prognostic factor for OS. It is also an aspect in the Revised Tokuhashi scoring system [16]. More than one vertebral segment usually reflect aggressive biological behavior and result in severe pain and spinal instability. Frankel score, KPS Score, ECOG Score partly reflect the neurological status of the patient neurological status.

HALP is a novel index combining hemoglobin and albumin levels and lymphocyte and platelet counts. It has been found significantly associated with outcomes in colorectal and gastric cancer [17, 18].Peng D et al [19]found that HALP was closely associated with clinicopathologic features and was an independent prognostic factor of cancer-specific survival for RCC patients undergoing nephrectomy. In our study, patient with HALP ≤ 20.67 was associated with poorer OS. But HALP only impact on OS in the univariate analysis, not multivariate analysis. These may be related to the small sample size relatively small sample size.

There are several limitations to our current study that include the retrospective data collection, the selection bias inherent to studies of this design, and the relatively small sample size. Despite these limitations, this study shows that for selected patients, surgical intervention can potentially prolong survival.

## Conclusions

RCC with visceral metastasis and AJCC RCC Stage were independent prognostic factors for SMFS. WHO/ISUP Grade, ECOG Scores and multiple spinal metastases were independent prognostic factors for OS.

## Data Availability

Please contact the corresponding author for data requests.

## List of abbreviations

RCC: Renal cell carcinoma
TD-ROC: Time-dependent ROC
NLR: Neutrophil-to-lymphocyte ratio
PLR: Platelet-to-lymphocyte ratio
AGR: Albumin-to-globulin ratio
LMR: Lymphocyte-to-monocyte ratio
HALP: The hemoglobin, albumin, lymphocyte, and platelet
KPS: Karnofsky Performance Status
TES: Total en-bloc spondylectomy
SRE: Skeletal-related events
MESCC: Metastatic epidural spinal cord compression
NESMS: New England Spinal Metastasis Score
OS: Overall survival
AJCC: Union International Against cancer
